# Distinctive Nuclear Features of Dinoflagellates with A Particular Focus on Histone and Histone-Replacement Proteins

**DOI:** 10.3390/microorganisms6040128

**Published:** 2018-12-14

**Authors:** Sadaf Riaz, Zhenghong Sui, Zeeshan Niaz, Sohrab Khan, Yuan Liu, Haoxin Liu

**Affiliations:** 1 Key Laboratory of Marine Genetics and Breeding (Ocean University of China), Ministry of Education, Qingdao, 266003, China; sadafriaz91@yahoo.com (S.R.); themicrobiologist@hu.edu.pk (Z.N.); khanhr39@yahoo.com (S.K.); liuyuan@ouc.edu.cn (Y.L.); lhx@stu.ouc.edu.cn (H.L.); 2 Department of Microbiology, University of Veterinary and Animal Sciences, Lahore 54000, Pakistan; 3 Department of Microbiology, Hazara University, Mansehra 21120, Pakistan

**Keywords:** Dinokaryon, chromatin, histones, histone-like proteins, dinoflagellate/viral nucleoproteins, evolution

## Abstract

Dinoflagellates are important eukaryotic microorganisms that play critical roles as producers and grazers, and cause harmful algal blooms. The unusual nuclei of dinoflagellates “dinokaryon” have led researchers to investigate their enigmatic nuclear features. Their nuclei are unusual in terms of their permanently condensed nucleosome-less chromatin, immense genome, low protein to DNA ratio, guanine-cytosine rich methylated DNA, and unique mitosis process. Furthermore, dinoflagellates are the only known group of eukaryotes that apparently lack histone proteins. Over the course of evolution, dinoflagellates have recruited other proteins, e.g., histone-like proteins (HLPs), from bacteria and dinoflagellates/viral nucleoproteins (DVNPs) from viruses as histone substitutes. Expression diversity of these nucleoproteins has greatly influenced the chromatin structure and gene expression regulation in dinoflagellates. Histone replacement proteins (HLPs and DVNPs) are hypothesized to perform a few similar roles as histone proteins do in other eukaryotes, i.e., gene expression regulation and repairing DNA. However, their role in bulk packaging of DNA is not significant as low amounts of proteins are associated with the gigantic genome. This review intends to summarize the discoveries encompassing unique nuclear features of dinoflagellates, particularly focusing on histone and histone replacement proteins. In addition, a comprehensive view of the evolution of dinoflagellate nuclei is presented.

## 1. An Overview of Dinoflagellates

Dinoflagellates are unicellular and motile eukaryotic microorganisms found in both freshwater and marine ecosystems [1]. Two flagella, transverse and longitudinal, are present in dinoflagellates and nearly 2/3 of the dinoflagellates possess a unique cell covering known as theca. The size of dinoflagellates varies from a few µm to 2 mm [2] and they exhibit a diversified life style, from free living to parasitic or symbiotic. More than 4500 species have been documented, among which nearly 4000 species are free living (class Dinophycea) whereas 40 are intracellular parasites (class Syndinea) [2]. Approximately 50% of dinoflagellates are photosynthetic [3], in addition to heterotrophic and mixotrophic type, and is one of the most important primary producers in marine and freshwater ecosystems. Symbiotic dinoflagellates, such as *Symbiodinium*, are essential for the formation of reef ecosystems whereas toxic dinoflagellate species produce harmful algal blooms (HABs) [4]. Dinoflagellates form an important group of plankton that have been present since the Jurassic period. They possess an extensive fossil record because of their ability to form resistant cysts [5]. Dinoflagellates can reproduce both sexually and asexually. The proliferation of photosynthetic dinoflagellate cells is slower compared to that of other phytoplanktons [6]. The less efficient rubisco of dinoflagellate evolved from anaerobic proteobacteria [7]. Low chlorophyll to carbon ratios are factors for their slower growth [8]. 

Dinoflagellate species are the most abundant among HAB samples [9] and are thus a major cause of HABs production. Conditions favoring HABs include increased nutrient availability (nitrogen, phosphorus, and trace elements), temperature, salinity, and other biological factors [10]. HAB species produce toxins; for example, Alexandrium species produce neurotoxins called paralytic shellfish toxins [11]. For some toxins, a dose at the microgram per kilogram level is more than enough to kill; the consumption of one or two contaminated mussels can kill a healthy adult human [12]. HABs are associated with massive fish mortalities, human and marine mammal intoxications, as well as economic losses. Nontoxic microalgal blooms are fatal to macrofauna as they cause bottom-water hypoxia [9].

Dinoflagellates possess a number of unusual features, e.g., unique plastids acquired from red algae via endosymbiosis [3,13,14], mitochondrial genomes encoding three proteins for the electron transport chain, and fragmented genes for rRNA [15,16]. It is also commonly accepted that the nuclei of dinoflagellates are particularly unusual. These are referred to as dinokaryon [17].

## 2. Distinctive Features of Dinokaryons

### 2.1. Genome Size and DNA Structure

Dinoflagellate nuclei contain large amounts of DNA and, with few exceptions, are haploid in their vegetative stage. Dinoflagellates contain 3 pg to 250 pg of genomic DNA [17], 1–83 fold that of human. The amount of DNA in other eukaryotes ranges from 0.04 pg to 3 pg (with some plants at 40 pg) [18]. However, genome size calculation largely depends on the compound used for measurement during flow cytometry [19]. For example, the Lingulodinium polyedrum genome size is reported as 200 pg [20] and 59 pg [19] using 3, 5-diaminobenzoic acid dihydrochloride and 4′, 6-diamidino-2-phenylindole dihydrochloride, respectively. The number of chromosomes in dinoflagellates varies from 4 to 270 [17]. Free living dinoflagellates possess more chromosomes, as high as 270, whereas parasitic dinoflagellates contain less chromosomes, as low as 4. Different types of substitutions are present in the DNA of dinoflagellates; 5-methylcytosine, N6–methyladenine, and 5-hydroxymethyluracil [21,22,23] have a random distribution in the genome [24]. During genome replication, 5-hydroxymethyluracil replaces 12% to 68% of thymidine and the presence of methylated DNA is predicted to be associated with gene expression regulation [25]. Some bacteriophages also possess this rare base and its function is to protect them from the restriction enzymes of the host [3].

Genome analysis shows that a high amount of non-coding DNA is present in dinoflagellates [26]. *Symbiodinium minutum* contains nearly 18.6 introns per gene whereas some contain more than 200 introns [27]. Many other studies have predicted few introns in dinoflagellates [28,29,30]. The amount of tandem repeat sequences varies from species to species, e.g., *Alexandrium ostenfeldii* genomes contain 58% [31] whereas ~10% of the genome in *S. minutum* [27] and ~9% of the genome in *S. kawagutii* is occupied by tandem repeat sequences [32]. 

The size of the genome correlates with the cell size, e.g., diatoms have a large cell size as their genome is correspondingly large (50 pg); the median cell size of dinoflagellates is nearly three times larger than that of diatoms [33,34]. Growth rate of the diatoms is two to three times faster than dinoflagellates; this can explain the difference in median dinoflagellate and diatom size [35]. In the Mesozoic era, both dinoflagellates and diatoms experienced changes in their cell size parallel to increasing temperatures, reaching their maximum size in the Eocene period (later in the Cenozoic period, the climate was cooled). This implies the role of climate change in the determination of cell size. It also implies that the stratification of ocean and nutrient availability leads to a larger genome as an adaptive response [33,36]. However, the exact reasons for the enlarged genomes in dinoflagellates are still unclear. Complete genomic data from different dinoflagellate species could aid in our understanding of the details, but gigantic genome sizes and higher costs are hurdles.

### 2.2. Nucleofilaments 

In eukaryotes, DNA (2 nm) is wrapped around the octamer of histone proteins (10 nm) resembling beads on a string called nucleosomes [3]. Nucleosomes are the basic units of nucleofilaments. Linker histones attach two nucleosomes, which further compacts the DNA into a 30 nm fiber [19]. Dinoflagellates are an exception to this rule because they do not bear nucleosomes. Nucleofilaments isolated from eukaryotes have a diameter of 10 nm, whereas nucleofilaments of dinoflagellates are a smooth thread with a diameter of 6.5 nm (thicker than normal DNA’s 2 nm diameter) [3]. Miccrococcal nucleases cut their DNA randomly unlike other eukaryotes, as their DNA is protected and cut into discrete lengths [37,38]. However, micrococcal endonucleases may digest nearly 10% of the DNA of dinoflagellates. Such observations imply that the DNA of dinoflagellates may be tightly bound by non-histone proteins. This compact conformation may protect DNA from enzymatic digestion [39]. However, it is unclear whether this protection is due to proteins or some other phenomenon. 

### 2.3. Transcription 

Dinoflagellates contain highly methylated and condensed DNA. Additionally, the presence of specific promoter and transcription factors requires a different gene expression mechanism. The chromatin of dinoflagellates remains condensed throughout the cell cycle [40]. This conformation is not feasible for transcription. Studies have proposed a model for transcription in dinoflagellates [41]. Left handed helices (Z-type DNA) are predominantly located at chromosomal margins and transcription may occur at the periphery [42]. Z-type DNA can be formed in condensed DNA. It detaches the packed DNA strings, creating coding regions available for transcription [42]. Chromosomes with ruffled boundaries and protruding loops from the periphery during the G1 phase allow maximum transcription during this phase. The absence of loops during the G2 phase suggests reduced transcription during division [2].

Spliced leader RNA (a short RNA sequence) is required for transferring at the 5’ of the newly transcribed mRNA to form mature mRNA [43]. This trans-splicing also occurs in other phyla, such as cnidaria and flatworms. However, this phenomenon is noteworthy in dinoflagellates due to some unique features; for example, its sequence is highly conserved among dinoflagellates and it is organized in tandem repeats [3]. The mRNA recycling hypothesis explains the occurrence of tandem repeats according to which mRNA is transcribed back to cDNA and integrated into the genome [44,45]. Plausible roles of trans-splicing are to regulate gene expression, make mRNA from polycistronic mRNA, and sanitize the mRNAs [46,47,48]. 

In dinoflagellates, TATA box is found to be absent in the promoter region of genes [49] and some mRNA do not contain poly-A tail [50]. TTTT is speculated to serve as a core promoter motif replacing the TATA [32]. The TATA box-binding protein is identified in dinoflagellates, which preferentially attaches to TTTT rather than the TATA box [51]. The exact mechanism of gene expression regulation in dinoflagellates without apparent histone proteins is unclear, though highly methylated DNA is suggested to be involved in gene expression regulation [25]. In addition, nuclear matrices are proposed as being involved in RNA synthesis and the organization of active DNA loops, thus regulating the gene expression [52]. Nevertheless, gene expression regulation at transcription levels has a reduced role in dinoflagellates [53,54]. The protein expression is mostly controlled at the post-transcriptional and translational level. Currently, a nuclear protein profile of a dinoflagellate species also strengthens this view by finding more RNA-binding proteins than DNA-binding proteins [55]. In addition, very few genes are proposed to have a sequence-specific transcription control in *Symbiodinium minutum* [27]. Transcriptome data from different species also show that a very small portion of transcriptomes respond to the diverse conditions [56,57,58,59,60] suggesting the post-transcriptional regulation of gene expression in dinoflagellates. Furthermore, in *Symbiodinium,* a large amount of intraspecific gene duplications represents an alternate mechanism of gene expression regulation [61].

When interpreting gene expression results, it is important to remember that transcript abundance is dependent on the rate of transcription and on the transcript degradation [62]. On the other hand, dinoflagellates are a diverse group of eukaryotes and contain divergent properties, particularly with respect to the possession of different DNA-binding proteins with an altered protein sequence and expression in different species. This could influence the transcription regulation differently between dinoflagellate species. 

### 2.4. Chromatin Condensation and Stabilization

In interphase cells, a complex of DNA, RNA, histone, and non-histone proteins is known as chromatin, or chromosomes in dividing cells [1]. Permanently condensed immense genomes and very low amounts of protein associated with the genome are an indication of specialized mechanisms of genome compaction and stabilization in dinoflagellates. Generally, at low concentrations, DNA is present in a dissolved form. At high concentrations, DNA can assemble to make liquid crystals. Formation of the liquid crystalline state is not only dependent on DNA concentration, but is also influenced by the solvent counterions [63]. Liquid crystalline states of DNA are found in viruses [64], bacteria [65], spermatozoa [66], and in dinoflagellates [67]. 

Large amounts of metal cations are present in the chromatin of dinoflagellates, which engage to maintain the chromosome structure [68]. Metal cations act to neutralize the negative charge of the phosphate groups in the DNA. Mg^2+^ and Ca^2+^ are also associated with other eukaryotic chromatins, but a higher concentration is reported in dinoflagellates. In addition to metal ions, chromatin RNA also plays an important role in chromatin packaging and stabilization. A study by Soyer in 1974 [69] shows that the treatment of chromatin with ribonuclease can loosen the chromatin and cause a threefold increase in length. However, when chromosomes are treated with EGTA (ethylene glycol-bis(β-aminoethyl ether)-N,N,N′,N′-tetraacetic acid) and KCL (potassium chloride) to remove proteins, this results in a complete disorganization of chromosomes [69]. Actin makes a network in dinoflagellate chromatin and supports the role of nuclear actin proteins in chromatin remodeling [70]. Moreover, the presence of a rare base (5-hydroxymethyluracil) in the DNA of dinoflagellates also contributes to condensing the gigantic genome [24]. Conclusively, proteins, RNA, and metal cations are required for compaction and stabilization. An increased genome size in a single-celled organism does not mean that the whole genome will code for proteins, however, the condensed genome may have a structural role, which is still unclear. Although dinoflagellates do not make nucleosomes for chromatin condensation, their chromosomes possess structural and functional differentiation similar to other eukaryotes [18]. Topoisomerases have also been detected in dinoflagellates [3,18] and their role in DNA condensation is very important. Dinoflagellates have type II topoisomerase activity in both the G1 and G2/M phases [71]. Recently, type I topoisomerase has been identified and it is proposed that histone-like proteins (HLPs) modulate its activity in dinoflagellate nuclei [72]. Large numbers (189) of genes for RCC (regulator of chromosome condensation), which are proposed as being involved in DNA condensation and gene expression regulation, are found in the dinoflagellate, *Symbiodinium minutum* [27]. This can provide another explanation for the immense genome condensation without nucleosomal organization. 

### 2.5. Cell Division 

Similar to other eukaryotes, dinoflagellates have discrete cell cycle phases; G1, S, and G2/M phases [73]. In photosynthetic dinoflagellates, the cell cycle is controlled by circadian rhythms and cell mitosis occurs at the end of the dark phase [6]. Most dinoflagellates are very sensitive to any turbulence; cell agitation activates bioluminescence in some of them [74]. The agitation of cells could result in cell cycle arrest at any stage [75], which is why harmful algal blooms appear when sea water is static. The uncommon process of mitosis in dinoflagellates is known as dinomitosis. Dinomitosis is a closed mitosis where nuclear envelopes persist throughout the cell cycle. This change is potentially the first step for the development of liquid crystalline chromatin, as this state of DNA is very sensitive to physiochemical changes. This may explain why dinoflagellates are sensitive to slight agitation and halt their division in case of turbulence in order to maintain genome integrity [6]. 

Dinomitosis is a specialized process of cell division because the chromosome remains condensed throughout the cell cycle, the nuclear envelope does not disappear during mitosis, the chromosome remains attached to the inner side of the nuclear envelope, the extranuclear spindles without direct contact with chromosomes are formed, and chromosomes do not possess kinetochore structures [3]. At the start of mitosis, chromosomes are transformed into Y/V shaped structures [76]. Cytoplasmic tunnels are formed, which penetrate through the nucleus during metaphase. Chromosomes, which are attached to the nuclear envelope, bind to spindle microtubules present inside the cytoplasmic tunnel. Nuclear membranes become thicker and denser where chromosomes are attached by telomeres [2]. Y/V shaped chromosomes then become separated, forming two disc-shaped daughter nuclei. After the completion of karyokinesis, the nucleus returns to a globular shape [76]. Distinct chromosomes are visible throughout cell cycles; however, they become slightly less condensed during different stages of the cell cycle, reaching maximum (and transient) unwinding during the DNA synthesis phase. Most condensed chromosomes are observed during the G2 phase [77]. 

## 3. Nuclear Proteins of Dinoflagellates

The protein-to-DNA ratio in other eukaryotes is 1:1 and 1:1.25 in prokaryotes [78], and 1:10 in core dinoflagellates [38]. However, in primitive dinoflagellates, (*Oxyrrhis* and *Hematodinium*), this ratio is 1:2 [37]. Nuclear proteins can be divided into two groups: Histone and non-histone proteins. Although histone proteins are better understood than non-histone proteins, a number of facts about them are presently unclear. 

### 3.1. Non-Histone Proteins of Dinokaryon

Most of the proteins present in dinokaryons are non-histone proteins. Non-histone DNA-binding proteins of *Crypthecodinium cohnii* have been proposed as being involved in cell cycle progression [79,80]. Several known non-histone proteins of dinokaryon (and their proposed functions) are dinoflagellate nuclear-associated proteins of *C. cohnii* (transcription regulation) [81], lamins protein of *Amphidinium carterae* (nuclear structure maintenance) [82], topoisomerase II of *C. cohnii* (transcription regulation) [71], TATA box-binding protein of *C. cohnii* (capable of binding TTTT promoters and may regulate transcription) [51], the regulator of chromosome condensation protein of *S. minutum* (chromosome condensation) [27], and the proliferating cell nuclear antigen of *Pfiesteria piscicida* (DNA replication) [83]. 

Whole cell acid-extracted proteins are sequenced through mass spectrometry (MS) in *A. pacificum*. This report identifies only three non-histone nuclear proteins carrying a DNA-binding domain [84]. The total nuclear protein profile of *L. polyedrum* was recently presented [55]. In this study, total nuclear proteins were sequenced by MS, which revealed 1245 nuclear proteins. Only 2.1% (26 proteins) of the total nuclear proteins were found to contain a DNA-binding domain and most of them were cold shock proteins. However, proteins carrying RNA-binding domains (108 proteins) were 10-fold more than the DNA-binding protein, implying that RNA-binding proteins may have a role in transcription regulation and structure maintenance of chromatin in dinoflagellates. 

### 3.2. Histone and Histone-Replacement Proteins of Dinokaryons 

**Figure 1:**
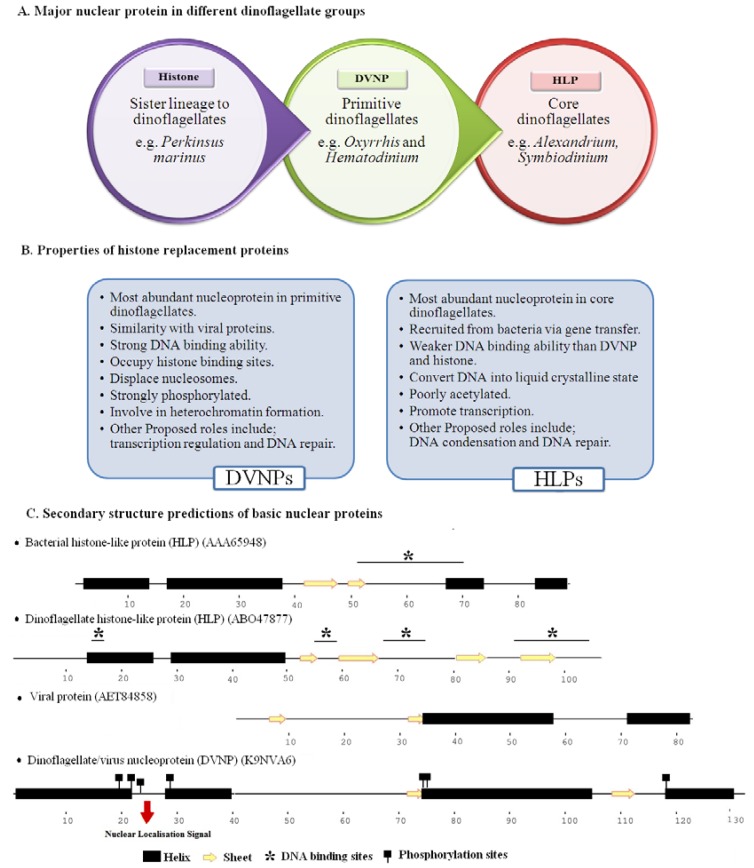
Histone replacement proteins; dinoflagellate/viral nucleoproteins (DVNP) and histone-like proteins (HLP) of dinoflagellates. (**A**) Most abundant nuclear protein in different dinoflagellate groups. (**B**) Characteristics of DVNPs and HLPs of dinoflagellates. (**C**) Secondary structure predictions of HLP and DVNP of dinoflagellates alongside bacterial HLP and viral protein. Representative protein sequences were downloaded from NCBI and uniprot while secondary structures were predicted using an online resource (http://bioinf.cs.ucl.ac.uk/psipred/) [90].

Dinoflagellates were previously thought to be histone-less. Other alkaline nucleoproteins, such as histone-like proteins (HLPs) [85,86,87,88] and dinoflagellate/viral nucleoproteins (DVNPs) [37], were determined as histone substitutes. Therefore, in this review, both types of proteins are described as histone-replacement proteins (Figure 1). Recent studies have shown the presence of histones at both the gene [89] and protein levels [37,55,84]. 

#### 3.2.1. Histone Like Proteins (HLPs) of *C. cohnii* Known as HCc

Ris (1962) [91] and Dodge (1964) [92] provided the first evidence that histone proteins are absent in dinoflagellates, using cytochemical analysis. On the other hand, Steward (1967) suggested the presence of histone proteins in dinoflagellates through an immuno-detection technique [93]. Cytochemical and immunochemical analyses are powerful techniques and produced conflicting results in this case. However, the antigen used by Steward (1967) was a DNA-protein complex rather than pure histone protein; therefore, this positive reaction is attributed as the HLP or non-histone proteins attaching to chromatin. Later, Rizzo (1972) solved this conflict by detecting HLP in dinoflagellates [94] and referred to this protein as HCc due to its isolation from *C. cohnii* [95]. HCc comprises 80% of the total acid-soluble proteins of chromatin [96] and HCc is the most extensively studied basic nucleoprotein of dinoflagellates.

HCc has a molecular weight of 16 kDa and its amino acid composition is different from that of typical histones [97]. The expression of HCc proteins changes between log and stationary phases. On the contrary, the expression of canonical histone protein does not change with increased metabolic activity [98]. In addition, HCcs are not conserved proteins, like histone proteins [95]. A 2-D SDS-PAGE revealed three different variants of HCc [99], and the structures of HCc1 and HCc2 were found to be remarkably different from core histone and other histone-like proteins. Surprisingly, these proteins share similarity with linker histones. HCc1 and HCc2 share 27% and 38% similarity with duck H5 [100]. HCc3 expression during the cell cycle is opposite to that of canonical histone proteins. HCc3 expression decreases during the S phase and reaches the maximum at the G2 phase, compared to canonical histones whose expression reaches the peak during the S phase and decreases in the G2 phase [72].

HLPs of bacteria and dinoflagellates present continuity in the evolution of eukaryotic linker histones from the bacterial HU family [101]. Despite the structural resemblance of HCc3 with bacterial HLP, functionally, the structure seems to be different. The DNA-binding ability of HCc is poorer than in bacterial HLPs. Furthermore, HCc3 cannot complement the bacterial HLP-deficient mutant because HCc3 does not have the ability to circularize DNA as bacterial HLPs do [85]. Functionally, HCc3 seems to be more similar to other DNA-binding proteins, known as H-NS-like proteins [102,103]. Although their sequences show less homology, both proteins are proposed to act as DNA-bridging agents. H-NS-like proteins have the ability to bend DNA [104], whereas HCc3 does not. 

The chromatin of dinoflagellates remains condensed throughout the cell cycle and active loops from DNA are created in the dinoflagellates [62], as well as in prokaryotes [105], to facilitate transcription. HCc3 compacts the DNA in a concentration-dependent manner so that DNA will compact under a higher concentration of HCc3, while being relaxed under a lower one [85]. In this concentration-dependent manner, HCc3 can create or collapse active DNA loops required for transcription. HCc can attach to single-stranded DNA with more affinity than to double-stranded DNA. This also supports its role in transcription regulation, because transcription requires the separation of DNA standards [99]. Furthermore, immuno-localization of HCc using immuno-gold labeling was completed previously. The immunofluorescence strongly suggested that HCc are involved in gene expression regulation because they are present at the periphery of chromosomes, where active transcription occurs [99]. In addition, during the G2 phase of the cell cycle, when chromosomes of dinoflagellates are highly condensed, the increased expression of HCc predicts its role in DNA condensation, while the homogenous spread of HCc throughout the chromosome is observed during mitosis via immuno-localization. Therefore, it could be hypothesized that HCc are involved in maintaining chromosomal structure [86,100,106]. The ability of HCc to condense the genes suggests its application in gene therapy as a viral-free gene transfer agent [107]. Moreover, HCc3 can bind to nick DNA with a higher affinity than to double-stranded DNA, revealing its role in repairing DNA. The presence of HCc3 prevents the DNA from undergoing nuclease digestion [72]. The DNA binding, condensing, and bridging ability of HCc3 lies in its C-terminal, while the N-terminal modulates its activity [85].

RNA performs a special role in chromatin stabilization. RNA-binding proteins play a significant role in post-transcriptional gene expression regulation. Thus, the attachment of HCc3 with RNA is tested in vitro. A positive interaction is observed, which may suggest the functional significance of HCc3 in this regard [72]. In addition to the role of HCc3 in transcription, DNA condensation, and DNA repair, it is also proposed that it is involved in DNA supercoiling by modulating the efficiency of topoisomerase [72]. The activity of topoisomerase can be stimulated by histone H1 in eukaryotes [108], whereas HCc3 can do this in dinoflagellates [72]. HCc3 induces supercoils in plasmid DNA in vitro, converting it into a liquid crystalline state [107]. This phenomenon is an evolutionarily important step for dinoflagellates, as the recruitment of HCc could potentially lead to the development of a liquid crystalline state of chromatin.

HCc play a number of roles in dinoflagellates, which ensures a probable system to regulate its function. In histone proteins, different post-translational modifications are responsible for controlling histone function. Acetylation is responsible for reducing the positive charge of histone proteins, resulting in a loosened bond between negatively-charged DNA and positively-charged histone proteins, making DNA accessible for transcription [109]. On the other hand, histone methylation could lead to transcription inhibition or stimulation. The post-translational modifications of HCc are poorly understood mechanisms in dinoflagellates.

Overall, HCc could perform most of the functions in dinoflagellates as histone proteins do in other eukaryotes. This may be one of the reasons for the reduced function and expression of histone proteins in *C. cohnii*. Nevertheless, a number of studies have been conducted on HCc revealing their different functions, while very few studies on histone proteins in dinoflagellates have been conducted so far because their presence has only been confirmed quite recently. 

#### 3.2.2 Histone-Like Protein-HLPs

HLPs of different dinoflagellate species share nearly the same electrophoretic mobility [110]. However, HLPs are not conserved, like histones, and their DNA-binding ability is weaker than in histones and DVNPs [87]. A dinoflagellate species may possess multiple variants of HLP; their genes may contain introns, and be present in tandem repeats [27]. Mass spectrometric analysis of the total acid-soluble proteins revealed that HLP is the most expressed acid-soluble protein (32% of total) in the core dinoflagellate, *A. pacificum* [84]. 

HLPs are also observed in other alveolates, e.g., human parasites; however, their origin is different [86]. Phylogenetic analysis suggests that the origin of HLPs of dinoflagellates is from prokaryotes via gene transfer [85,86]. Previous phylogenetic analysis shows that HLPs originated from the same ancestor and later on diversified through speciation events [72]. However, recent studies indicate the origination of HLPs from at least two different prokaryotic ancestors [111]. HLPs form two different clades; HLP-II consists of an early core dinoflagellate species whereas HLP-I consists of a late-branched core dinoflagellate species. This study also proposed that HLPs first appeared in prokaryotes, compared with DVNPs in which the direction of transfer is not established yet. The DNA-binding ability [72] and protein sequences vary greatly between HLP-I and HLP-II groups regardless of their same protein size [111]. Similarly, less homology between HLPs obtained from dinoflagellate species shows that different dinoflagellate groups recruited HLPs from different bacteria via gene transfer [18]. 

In contrast to bacterial HLPs, HLPs of dinoflagellates are highly sequence-dependent proteins and a slight change or deletion can reduce or cease their designated function [72]. Their C-terminal contains a more conserved sequence compared to the N-terminal, which suggests the presence of putative regulatory domains [87,112]. A study carried out by Zhang (2014) wherein 48 HLPs from 15 dinoflagellate species were analyzed, found that a novel DNA-binding domain is present in all 48 HLPs and absent in bacterial HLP [72]. This domain is predicted to have evolved from the DNA-binding hairpin arm of bacterial HLP. Further analysis confirms that late branches of core dinoflagellates possess more evolved DNA-binding domains in their HLP and have increased DNA aggregation abilities compared to the early-branching core dinoflagellates [72]. The removal or replacement of amino acids from this domain decreases the DNA aggregation efficiency of HLP. HLP posttranslational modification (acetylation) is also observed, contributing to its role in gene expression [112]. 

The increased expression of HLP transcripts is observed in *Pyrocystis lunula* in f/2 medium containing 1mM sodium nitrite and predicted to be redox regulated [56]. This suggests a role of HLP during stress conditions. However, HLPs are not differentially expressed during nutritional stress [113] or heavy metal stress [114]. During the cell cycle, the expression of HLP in few dinoflagellates has been studied. During the G1 phase of the cell cycle, the expression of HLP in *P. lunula* [57] and *Alexandrium fundyense* [115] are found to be upregulated. The production of toxins is believed to be cell cycle-regulated. Peak toxin production in *A. catenella* occurs during the S phase [116], in *A. fundyense* during the G1 phase [117], and in *Alexandrium ostenfeldii* during the late G1 and S phases [118]. Transcriptome sequencing of *A. pacificum* indicates that the HLP transcript level increases under nutrient-enhanced cultures [119]. The availability of nutrients and trace elements is a contributing factor for bloom production. Therefore, studying HLPs during different growth conditions could shed light on their detailed roles in dinoflagellates. 

#### 3.2.3 Dinoflagellate/Viral Nucleoproteins-DVNPs

*Oxyrrhis* (a primitive dinoflagellate) gives a strong, positive reaction to alkaline fast green staining, which is used to stain basic nuclear proteins. In addition, nuclear acid extracts of *Oxyrrhis* present just one major band (23 kDa) compared to core dinoflagellates. Core dinoflagellates give negative alkaline fast green staining and produce multiple bands from nuclear extracts. As a major nuclear protein of *Oxyrrhis* was speculated as being different from HLPs, it was named Np23 and immune-localization confirmed its attachment to DNA and proposed its role in chromosome structure [120]. 

Recently, basic nuclear proteins from *Hematodinium* (another primitive dinoflagellate) have been found to produce one major band on gel similar to *Oxyrrhis* [37]. MS analysis of this protein has revealed a different protein from HLP, which has been named as dinoflagellate/virus nucleoprotein (DVNP) as its sequence shares similarity with viral proteins [37]. Np23 is considered to be a DVNP in the same report as both share similar properties. Staining cells with alkaline fast green gives strong reactions in these two primitive dinoflagellates, whereas the same staining does not produce any reaction for core dinoflagellate species. This suggests a higher expression of basic nuclear proteins in primitive dinoflagellates.

DVNPs are highly basic and abundant nuclear proteins compared to HLPs or histones of dinoflagellates. Different variants of DVNPs have been identified, 13 (molecular size from 13.4 kDa to 20.2 kDa) in *Hematodinium* [37]*,* 20 in *Oxyrrhis marinus* [121], and 19 in *Symbiodinium minutum* [27]. The number of DVNP variants are greater than HLP in dinoflagellates, e.g., *Alexandrium pacificum* contains only two variants for HLP [122] while *Alexandrium monilatum* contains 38 variants for DVNPs (highest among all dinoflagellate species) [89]. A set of homologs, DVNP1 and DVNP10, is present in *S. minutum* in tandem. DVNP1 is intron-less while DVNP10 contains multiple introns [52]. The alternative splicing of introns may be responsible for the production of different isoforms from the same gene [123]. Gene duplication could be another reason for multiple variants; however, it appears that DVNP genes are located on different assembly scaffolds in *S. minutum* [27]. Similarly, it is not clear how 19 different homologs could be introduced into genomes at different places if they are recruited via gene transfer or endosymbiosis [52]. 

DVNP gene sequences are reported in all tested dinoflagellate species; however, no other eukaryote or prokaryote is reported to have this [37]. The expression level of DVNPs differs between species, e.g., primitive dinoflagellates show a higher expression of DVNP and lack HLP, while core dinoflagellate shows less expression of DVNPs and higher expression of HLP. As well as the expression level, the conservation level of DVNP also varies greatly between dinoflagellate species. *S. minutum* contains 19 genes for DVNP; however, deduced proteins of 11 genes contain additional domains that are absent in primitive dinoflagellates, suggesting the divergent role of DVNP in primitive and core dinoflagellates [27]. 

Phylogenetic analysis supports the common origin of viral proteins and DVNPs [111]. It is not clear whether DVNP appears at first in dinoflagellates or in viruses. Gorink (2012) proposed a possible evolutionary event, suggesting that ancestors of dinoflagellates became infected with viruses and received this gene via lateral gene transfer. It is, however, quite impossible that DVNPs detected in dinoflagellates are the products of viral infection, as their sequences contain splice leader sequences and the presence of multiple DVNP genes rejects this idea. In addition, it also contains nuclear internalization sequences in its N-terminal (absent in viral proteins) (Figure 1) [37]. 

DVNPs occupy histone-binding sites and displace nucleosomes [124], causing a reduction in histone expression in dinoflagellates. Abundant DVNPs and a poorly expressed histone protein, H2A, have been reported in *Hematodinium* [37]. DVNPs are proposed as DNA-bound post-translationally modified (highly phosphorylated) proteins and their DNA-binding ability is equal to that of histone proteins [37]. Conversely, HLPs do not bind to DNA with this efficiency and are poorly acetylated [112]. DVNPs are proposed as being involved in heterochromatin formation, as most of the histone marks required for DNA condensation are absent in dinoflagellates [124]. A study was carried out to reveal the role of DVNP in *C. cohnii* (although this dinoflagellate has HLP as a major nuclear protein) [52]. The study suggested that the DVNP of this species is involved in transcription regulation and its role is also hypothesized during DNA damage and osmolality stress. However, no significant change was observed during temperature stress in this study. During DNA damage, the expression of HCc3 and DVNP was found to be up-regulated. However, an increase in DVNP expression was observed as being higher than HCc3 in the same species. The expression of DVNP during the cell cycle is opposed to the expression of typical histone proteins. Its transcript expression remains stable during the G1 and S phase and reaches its peak during the G2 phase, supporting its role in DNA condensation. However, HCc3 expression levels were more increased than DVNP in *C. cohnii* during the G2 phase [52], suggesting a more significant role of HLPs in condensation than DVNPs in core dinoflagellates. 

DVNPs are the most expressed basic nuclear proteins of dinoflagellates, possessing strong DNA binding and posttranslational modification abilities. 

#### 3.2.4 Histone Proteins

The study of histone proteins in dinoflagellates is almost a negligible area of research compared to other eukaryotic histone proteins. The very first evidence of the presence of histone genes was found in 2003 [56]. Since then, different transcriptomic studies have identified different histone genes in multiple dinoflagellate species [27,48,119,125,126,127]. However, there have been only two reports on the successful detection of expressed histone proteins of dinoflagellates by mass spectrometry: H2A [37], H4 [55], and one report on the immuno-detection of histone proteins (H2B and H4) [84]. Still, we have attempted to focus on stimulating the interest of researchers in this overlooked field. 

**Figure 2:**
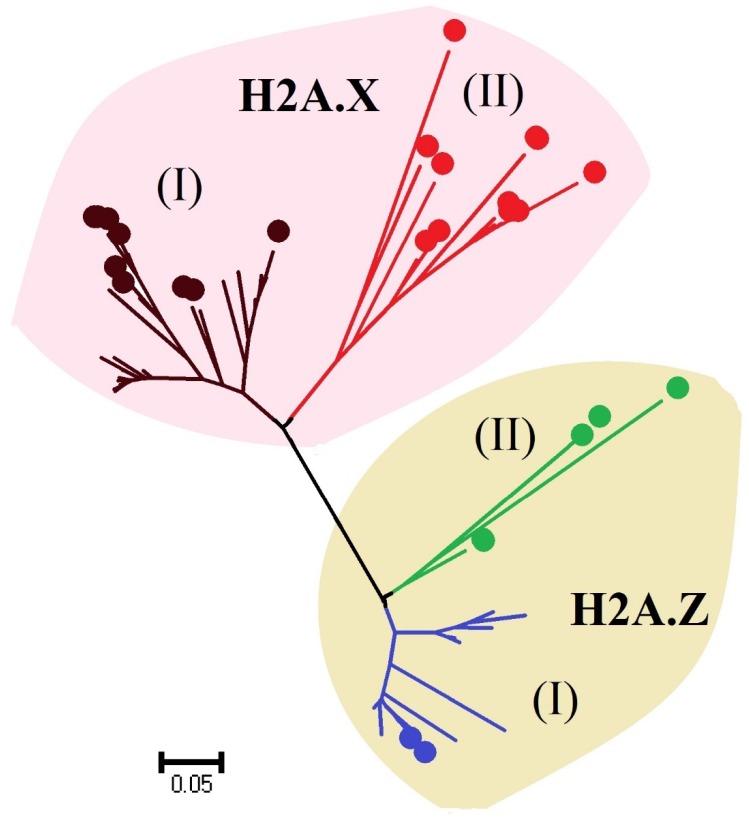
Neighbor-joining (NJ) phylogenetic tree of H2AX and H2A.Z variants. Phylogenetic analysis was performed using the software, MEGA 5.0 [131]. Total full length protein sequences (75) used in this analysis were downloaded from NCBI and MMETSP (http://marinemicroeukaryotes.org) [132]. Colored circles represent protein sequences from dinoflagellates. In (I), histones of dinoflagellates are grouped with other eukaryotes while (II) contains the dinoflagellate-specific histone variants.

Different variants of histone genes have been identified in multiple species of dinoflagellates. Some of them exhibit sequence similarity with other eukaryotic histones, while other variants are divergent [89]. However, this sampling is based on transcriptome data and it is quite possible that transcriptome data do not present a complete set of variants present in a species, because of the absence of poly-A tail in canonical histones. Phylogenetic analyses of *A. pacificum* and *L. polyedrum* suggest that these species contain at least two variants for each histone type, one grouped with eukaryotic histones while the other is a dinoflagellate-specific histone [84,126] (Figure 2). Histone proteins share different functional motifs, e.g., the H2AX variant contains a functional motif (SQEF/SQEY) in the C-terminal, which is involved in repairing DNA [128]. H2AX and H2AZ variants of H2A are reported in dinoflagellates (Figure 2). Similarly, different variants of H3, H3.1, and H3.3 are present in dinoflagellates [89], suggesting the role of histones in transcription regulation. H3.1 corresponds to the genome-silenced areas whereas H3.3 is present in transcriptionally-active regions [129,130]. Most dinoflagellate species contain the H3.3 variant while only a few species possess the H3.1 variant, suggesting that histones correspond to the euchromatin in dinoflagellates.

The possibility of histone genes in the transcriptome of dinoflagellates due to contamination may be neglected. This is because divergent histone sequences are identified in dinoflagellates and histone sequences sometimes contain splice leader sequences. Above all, axenic and non-axenic cultures exhibit histone genes [89].

**Figure 3:**
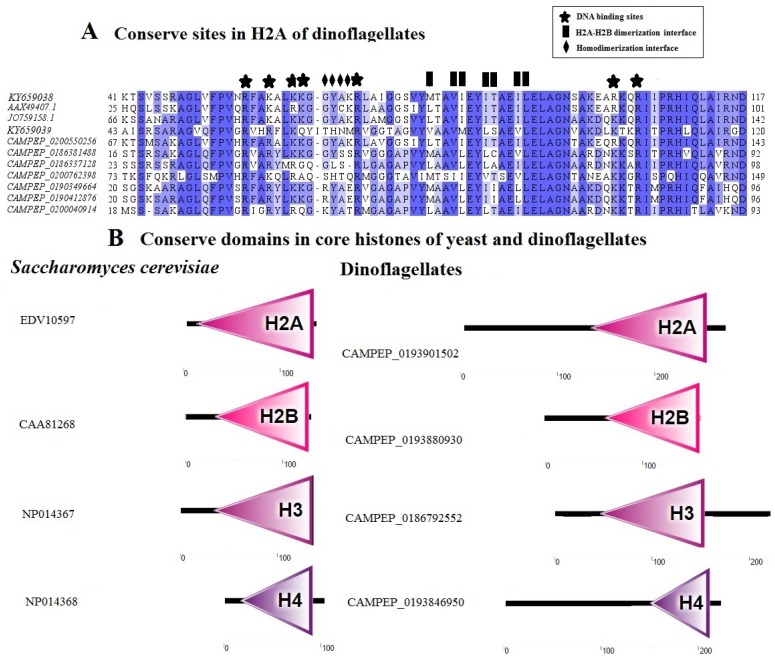
(**A**) Conserved sites in H2A of dinoflagellates. Conserved sites are predicted using an online resource (http://www.ncbi.nlm.nih.gov/Structure/cdd/wrpsb.cgi) [133]. (**B**) Conserved histone domains in yeast and dinoflagellates. These domains are predicted using a resource at http://smart.embl-heidelberg.de/ [134].

In eukaryotes, histone protein sequences are highly conserved and any change in a single nucleotide can bring about change in their DNA-binding or dimerization ability (H2A-H2B/H3-H4). The conserved sites required for DNA-binding and homo- or hetero-dimerization are present in the histones of dinoflagellates, which indicates the possibility of nucleosomes in dinoflagellates (Figure 3A) [84]. The elongated and divergent N-terminus is a distinguishing feature of dinoflagellate histones. Their deduced histone protein sequence analysis shows that N-terminal sequences are very divergent and elongated; however, core histone domains are conserved (Figure 3B). Sometimes, the elongated N-terminal is due to the presence of multiple start codons (methionine). It was previously proposed that they were not a part of the functional protein because they do not share any sequence similarity with other species or their own species’ variants [122]. Extended N- terminals of HLP are involved in DNA-binding and the N-terminal of DNVP contains a nuclear internalization sequence. However, the role of extended N-terminals in functional histone proteins needs to be elucidated.

The amino acid sequence conservation in histone tails is closely related to the functional significance of histones in dinoflagellates. Following this, the level of conservation of amino acids (histone code) in their protruding N-terminus is assessed [84,89]. The result of this analysis indicates that many amino acids are conserved in dinoflagellates. It is therefore hypothesized that these conserved residues may play a significant role in gene expression regulation and cell cycle progression. Histone proteins of dinoflagellates possess more conserved sites for transcription activation than heterochromatin-associated modifications. This suggests that histone proteins have more functions in genome active areas than heterochromatin formation [124]. From this analysis, it is also suggested that histone code is most conserved in the primitive dinoflagellate, *Oxyrrhis,* and in later species, their derived forms are present [89]. 

Conserved residues in the N-terminals of histones can be chemically modified by histone-modifying enzymes, which regulate the genetic information encoded in DNA. Dinoflagellates contain multiple histone-modifying enzyme-encoding genes [55,74]. It remains possible that the histone-modifying enzymes in dinoflagellates can target non-histone proteins [135,136,137,138]. HLPs and DVNPs of dinoflagellates are also targets for histone-modifying enzymes. Acetylation of HLPs [87] and phosphorylation of DVNPs are reported in dinoflagellates [37]. The detection and additional detailed analyses of histone-modifying enzymes will be important for our understanding of the role of histone proteins in dinoflagellates.

Antibodies of other eukaryotic origins may detect core histone proteins in yeast [126] and sister lineages of dinoflagellates *(Perkinsus marinus)* [37] because of conserved histone sequences and abundant protein availability. However, these same reports were unable to immuno-detect histones from dinoflagellate species. Histones show different transcript expressions in different species. Histone gene expression was reported as quite low in *L. polyedrum* [126] and rational expression is observed in *Azadinium spinosum* [139] and *A. pacificum* [84]. However, histone protein expression is quite low in dinoflagellates [122]. Accumulation levels of histone proteins are maintained at multiple levels, such as post-transcription and post-translation levels. It is speculated that histone genes are transcribed by the cell and are not translated sufficiently to form an easily-detected level, or translated proteins are enzymatically digested immediately. In research conducted by Beauchemin (2017), a total of nine samples were collected from a culture on the same day and H4 was detected in just one sample [55]. Lin (2010) proposed that histone expression is difficult to detect because these proteins are expressed under certain conditions, such as under some stress circumstances or during cyst formation [48]. However, a recent study showed that histone H4 expression is detectable under all tested conditions [84]. At the same time, H2B expression in this study is hardly detectable due to low expression. 

The expression of histone genes during the cell cycle remains stable during the S and G phases; thus, it belongs to the class of replication-independent genes [84,126]. Increased protein expression during high cell division cultures indicates that histone proteins have a significant role during algal bloom production [84]. Although it is accepted that histone expression is very low in dinoflagellates, it is still a matter of concern as small amounts of DNA-bound histone proteins can also regulate gene expression [140], as 0.05% to 1.8% of the genome codes for proteins in dinoflagellates [141]. Recently, a FACT (facilitates chromatin transcription) complex has been identified in different dinoflagellate species, suggesting that transcription through nucleosomes can occur in dinoflagellates [89].

After the successful immunological detection of histone proteins, it is also possible to immuno-localize the histone proteins during different stages of the cell cycle to uncover their roles during transcription and DNA condensation, using high quality dinoflagellate-specific histone antibodies. Efficient nuclear extraction and immuno-localization techniques have not been developed for most dinoflagellates (e.g., *Alexandirum* species due to their tougher cell wall), which poses a major hindrance for such analysis. Recombinant expressions of dinoflagellate histones, particularly the divergent type, could help to measure their in vitro effect on DNA condensation, chromosome remodeling, DNA-binding abilities, and modulating effects on other nuclear proteins. The role of histones in compacting smaller portions of DNA, transcription regulation, and repairing DNA requires further study.

**Figure 4:**
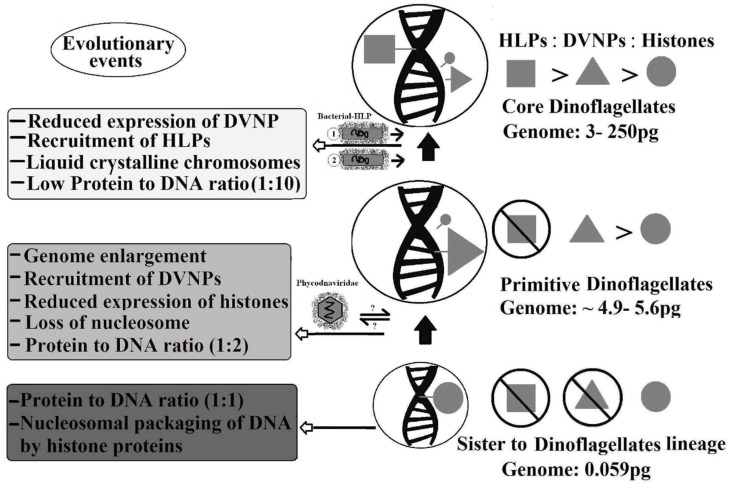
Model of dinokaryon evolution based on present evidence.

The first study on the detection of all three kinds of expressed histones and histone-replacement proteins in a core dinoflagellate species (*L. polyedrum*) has been published [55]. This species expresses 60 peptides for HLP, 15 peptides for DVNP, and two peptides for histone H4. This pattern (HLP > DVNP > histone) shows the expression difference of three kinds of basic nuclear proteins in core dinoflagellates (Figure 4).

## 4. Evolution of Dinokaryon, with Reference to Histones and Histone-Replacement Proteins

Endosymbiosis, lateral gene transfer, and gene duplication are major players driving evolution. Dinoflagellates received multiple genes through endosymbiosis. Individual genes are gained through lateral gene transfer, which plays a significant role in prokaryotic evolution [142]. Likewise, genes for different major nuclear proteins, obtained via lateral gene transfer, lead to the development of novel traits in dinoflagellate nuclei.

In order to answer how dinokaryon of core dinoflagellates evolved, it is necessary to study their relationship with their neighbor (Figure 4). Primitive dinoflagellates (*Oxyrrhis* and *Hematodinium*) have a genome size of 4.9 pg and 5.6 pg, respectively. On the other hand, the sister lineage of dinoflagellates, *Perkinsus marinus,* has a genome size of 0.059 pg [37]. This shows more than an 80-fold difference in genome size in primitive dinoflagellates compared to their sister lineage. This indicates that genome enlargement is an early step in the evolution of dinoflagellates. This increased genome leads to the recruitment of DVNPs in primitive dinoflagellates to condense their larger genomes [37]. It is proposed that dinoflagellates recruited DVNP from large algal viruses, most Phycodnaviridae species possess DNVP homologs, which then help to condense the large genome [35,36]. *Perkinsus marinus* possesses nucleosomes and abundant histone proteins, while primitive dinoflagellates lack nucleosomes, carry abundant DVNPs, and show a reduced expression of histones [37]. A recent study demonstrates that DVNPs are toxic to the cell at higher histone expressions [124]. Therefore, the acquisition of DVNP induces dinoflagellates to reduce histone expression as an adaptive response. This indicates a major evolutionary phenomenon coinciding with nucleosomal loss and the unique packaging of dinoflagellate DNA. Whether DVNPs appeared first in viruses or in dinoflagellates, it is clear that the recruitment of HLP is a later step in dinokaryon evolution, as primitive dinoflagellates do not contain HLPs.

The protein to DNA ratio in primitive dinoflagellates is 1:2, whereas in core dinoflagellates, it is 1:10. Hence, in the development of dinokaryon, increased genome size, DVNP acquisition, reduced histone expression, and nucleosomal loss occurred earlier on. The possession of HLPs, development of a liquid crystalline state of chromatin [111], and a low protein to DNA ratio are later events. Histone proteins are present throughout dinoflagellate evolution with a reduced expression and different levels of sequence conservation (more derived form in later branches) [89].

## 5. Concluding Remarks

Dinoflagellates are of particular interest because they are the most abundant and notorious species among HABs, thus a model for studying algal blooms. Dinoflagellates possess many novel features that make them significant; their nuclei, for instance, are truly deviant from typical eukaryotes. Non-eukaryotic features include nucleosome-less permanently condensed chromatin, increased GC contents, rare bases in DNA, low protein to DNA ratios, the presence of histone-like proteins, and closed mitosis. Nevertheless, discrete cell cycle phases, repetitive DNA sequences, functional nuclear organization, and the presence of histone proteins are all true eukaryotic characteristics. Over the course of evolution, dinoflagellates have recruited other proteins, e.g., HLPs, from bacteria (core dinoflagellates) and DVNPs from viruses (primitive dinoflagellates), as a major nuclear component. The expression diversity of these histone-replacement proteins has altered the chromatin structure and gene expression mechanism of dinoflagellates. The histone-replacement proteins are designated to perform crucial roles that cell-like histone proteins do in other eukaryotes. 

Histone proteins of dinoflagellates are not conserved proteins, like typical histone proteins, which share conservation over large scales and share multiple evolutionary origins. Notably, the presence of different variants of histone genes, conservation of histone code associated with transcription activation, and presence of different histone-modifying enzymes, supports histone functional significance in dinoflagellates. Dinoflagellate histone proteins possess conserved sites required for histone-DNA and histone-histone interaction. Moreover, genes required for nucleosome assembly and remodeling are also reported in multiple dinoflagellate species [89]. This evidence supports the idea that nucleosomes may be present in dinoflagellates, at least at the periphery of chromatin. Successful in vitro reconstruction of dinoflagellate nucleosomes also assists this view and provides evidence that highly methylated bases in DNA do not inhibit the formation of nucleosomes in dinoflagellates [143]. Nucleosomes can be disrupted during isolation due to their delicate nature as unstable nucleosome-like structures has been reported in *E. coli* [144] and a nucleosomal pattern from sperm cell nuclei is apparent under special isolation conditions [145].

Therefore, the apparent absence of nucleosomes and the difficulty of histone detection from dinoflagellate nuclei does not rule out their role in transcription. The detailed roles of histones, DVNPs, and HLPs in dinokaryon remain to be elucidated.
